# Evaluating correlation of ABO blood group, dental caries and gingivitis among the Indian population

**DOI:** 10.6026/973206300213088

**Published:** 2025-09-30

**Authors:** Kaushal Sharma, Prakhar Agarwal, Ananya Bhargava, Charu Khurana, Sufia Parveen, Chaitanya Bharathi Boya, Miral Mehta, Jugajyoti Pathi

**Affiliations:** 1 Department of Oral Medicine and Radiology, Daswani Dental College, Kota, Rajasthan, India; 2 Department of Oral Pathology and Microbiology, Darshan Dental College, Udaipur, Rajasthan, India; 3 Consultant Orthodontist, Ujjain, Madhya Pradesh, India; 4 Department of Public Health Dentistry, Faculty of Dental Sciences, SGT University, Chandu Budhera, Gurugram, Haryana -122505, India; 5 Department of Conservative Dentistry and Endodontics, Buddha Institute of Dental Sciences and Hospital, Patna, Bihar, India; 6 Department of Public Health Dentistry, G. Pulla Reddy dental college and hospital, Kurnool andhra Pradesh, India; 7 Department of Pediatric and Preventive Dentistry, Karnavati School of Dentistry, Karnavati University, Gandhinagar, Gujarat, India; 8 Department of Oral & Maxillofacial Surgery, Kalinga Institute of Dental Sciences, KIIT Deemed to be University, Bhubaneswar, Odisha, India

**Keywords:** ABO blood groups, dental caries, gingivitis, oral health, DMFT index, gingival index, Indian population, risk factors

## Abstract

Dental caries and gingivitis are highly prevalent worldwide, with limited evidence on the role of ABO blood groups in influencing susceptibility among Indians. A cross-sectional study was conducted on 600 adults, with blood groups determined serologically; caries assessed using the DMFT index and gingivitis using the Gingival Index. Blood group O showed the highest mean DMFT (4.82 ± 2.15) and gingivitis prevalence (82.6%), with significant differences across groups (p<0.01). Logistic regression identified group O as an independent predictor for high caries (OR=2.15) and gingivitis (OR=1.94). Blood group O may represent a non-modifiable risk factor for poor oral health, highlighting the need for targeted preventive strategies.

## Background:

Dental caries and gingivitis represent the two most common chronic diseases affecting humankind, imposing a substantial burden on individuals and healthcare systems worldwide [1]. Dental caries, a multifactorial disease resulting from the demineralization of tooth structure by acidogenic bacteria in dental plaque, leads to pain, infection and tooth loss [2]. Gingivitis, the inflammation of the gingival tissues induced by the accumulation of bacterial biofilm, is the precursor to periodontitis, which can ultimately destroy the supporting structures of the teeth [3]. The prevalence of both conditions remains high in India, with national surveys indicating caries experience affecting over 50% of adults and gingivitis prevalence exceeding 80% in many regions [4, 5]. This high burden underscores the critical need to identify and understand all potential risk factors, including genetic predispositions, to develop effective preventive and management strategies. The ABO blood group system, determined by specific oligosaccharide antigens on red blood cells and various epithelial cells, is one of the well-studied human genetic polymorphisms [6]. Beyond its crucial role in transfusion medicine, associations between ABO blood groups and susceptibility to various infectious diseases (*e.g.*, Helicobacter pylori, norovirus), inflammatory conditions (*e.g.*, cardiovascular disease, diabetes mellitus) and even certain cancers have been extensively investigated [7, 8]. The proposed mechanisms linking blood groups to disease susceptibility include modulation of the immune response, influence on inflammatory pathways, alterations in cell adhesion properties and variations in the composition of secreted glycoconjugates in body fluids like saliva [9, 10]. Saliva, a critical component of the oral environment, contains numerous glycoproteins and glycolipids whose expression can be influenced by ABO blood group status (secretor status) [11]. These salivary components play vital roles in microbial adhesion, colonization and the formation of the acquired enamel pellicle. Consequently, variations in salivary composition related to blood group could theoretically impact the oral microbiome ecology and the host's inflammatory response to plaque bacteria, thereby influencing caries and gingivitis risk [12, 13]. Several studies globally have explored the potential link between ABO blood groups and oral diseases, with findings often conflicting. Some studies reported an association between blood group O and higher caries prevalence or severity [14, 15], while others found no significant association or linked different blood groups (*e.g.*, A or B) to increased risk [16, 17]. Similarly, investigations into gingivitis and periodontitis have yielded inconsistent results; with some suggesting blood group O is associated with worse periodontal health [18, 19] and others finding no correlation [20]. A significant research gap exists regarding the relationship between ABO blood groups and oral health within the diverse Indian population. India's unique genetic admixture, varying dietary patterns, oral hygiene practices and socio-economic factors necessitate population-specific investigations. Most existing studies on this topic in India are either dated, limited by small sample sizes, or lack a comprehensive assessment of both caries and gingivitis using standardized indices [21, 22]. Furthermore, the potential interaction between blood group and other established risk factors like oral hygiene remains inadequately explored in this context. Therefore, it is of interest to rigorously evaluate the correlation between ABO blood groups and the prevalence and severity of dental caries and gingivitis among a representative sample of Indian adults, controlling for key confounding variables.

## Materials and Methods:

A cross-sectional study design was employed.

## Sample size calculation:

The sample size was calculated using the formula for cross-sectional studies comparing proportions. Assuming a gingivitis prevalence of 75% in the reference blood group (non-O) and expecting a 15% higher prevalence (90%) in blood group O based on preliminary literature review, with 80% power, 95% confidence level and accounting for an estimated blood group O distribution of 35% in the Indian population, a minimum sample size of 542 was required. Anticipating a 10% non-response rate or incomplete data, the final target sample size was set at 600 participants.

## Study participants and sampling:

A total of 600 consenting adults (300 males, 300 females) aged 18-60 years were recruited using a systematic random sampling approach. Every third individual attending the camp registration desk and meeting the inclusion criteria was invited to participate until the target sample was reached.

## Inclusion criteria:

[1] Adults aged 18-60 years.

[2] Willingness to participate and provide written informed consent.

[3] Presence of at least 20 natural teeth (excluding third molars).

[4] No history of systemic diseases known to significantly affect oral health (*e.g.*, uncontrolled diabetes mellitus, immunosuppressive conditions, blood disorders).

[5] Not pregnant or lactating.

[6] No antibiotic use within the past 3 months.

[7] No professional periodontal treatment (scaling/root planing) within the past 6 months.

## Exclusion criteria:

[1] Presence of orthodontic appliances.

[2] Presence of gross oral pathology (*e.g.*, large tumors, significant soft tissue abnormalities).

[3] History of head and neck radiotherapy.

[4] Inability to comply with the examination procedure.

## Data collection tools and procedures:

Data collection was performed by two calibrated examiners (inter-examiner kappa > 0.85 for both DMFT and GI) under standardized illumination using mouth mirrors, CPI probes and artificial light. Demographic and Behavioral Data: A structured questionnaire was administered to collect information on age, gender, education level, dietary habits (frequency of sugar consumption), smoking/tobacco use status and self-reported oral hygiene practices (frequency of brushing, use of adjuncts). ABO Blood Group Determination: Approximately 2 ml of venous blood was collected from the antecubital vein by a trained phlebotomist using aseptic technique. Blood grouping was performed immediately using commercially available standard tile method with anti-A, anti-B and anti-D (Rh) antisera (Tulip Diagnostics (P) Ltd., Goa, India). Results were recorded as A, B, AB, or O, along with Rh status.

## Oral health assessment:

## Dental Caries:

The DMFT index was used to assess caries experience. Each tooth was examined visually and tactilely (using a CPI probe gently). Decayed (D), Missing (M) due to caries and Filled (F) teeth were recorded according to World Health Organization (WHO) criteria [23]. The DMFT score was calculated as the sum of D, M and F teeth for each individual.

## Gingivitis:

The Gingival Index (GI) by Löe and Silness was used to assess gingival inflammation [24]. Four gingival areas (mesial, distal, buccal, lingual) around all teeth (excluding third molars) were examined. Scores were assigned: 0 = Normal gingiva; 1 = Mild inflammation (slight change in color, slight edema, no bleeding on probing); 2 = Moderate inflammation (redness, edema, glazing, bleeding on probing); 3 = Severe inflammation (marked redness and edema, ulceration, tendency to spontaneous bleeding). The GI score for each individual was calculated as the average score for all examined areas. Gingivitis was defined as GI > 0. Moderate/Severe gingivitis was defined as GI ≥ 1.1.

## Oral hygiene status:

The Simplified Oral Hygiene Index (OHI-S) by Greene and Vermillion was used to assess oral hygiene [25]. Debris and calculus scores were recorded on six predetermined tooth surfaces (16, 11, 26, 36, 31, 46). The OHI-S score was the sum of the Debris Index (DI-S) and Calculus Index (CI-S) scores.

## Statistical analysis:

Data were analyzed using SPSS software version 27.0 (IBM Corp., Armonk, NY, USA). Descriptive statistics (mean ± standard deviation (SD) for continuous variables, frequencies and percentages for categorical variables) were calculated for all variables. Normality of distribution was assessed using the Shapiro-Wilk test. Differences in mean DMFT, GI and OHI-S scores across blood groups were analyzed using one-way Analysis of Variance (ANOVA), followed by post-hoc Tukey's HSD test for pairwise comparisons.

## Results:

The study included 600 participants (300 males, 300 females) with a mean age of 35.7 ± 11.2 years. The distribution of ABO blood groups was: O (38.3%, n=230), A (28.7%, n=172), B (25.0%, n=150) and AB (8.0%, n=48). Rh positivity was observed in 94.5% of participants. There were no statistically significant differences in age, gender distribution, education level, or tobacco use across the blood groups (p > 0.05 for all). However, participants with blood group O reported significantly higher frequency of sugar consumption (≥3 times/day) compared to other groups (p=0.03) (Table 1 - see PDF). The overall mean OHI-S score was 2.85 ± 1.12. Mean OHI-S scores did not differ significantly across blood groups (O: 2.91 ± 1.15, A: 2.78 ± 1.08, B: 2.82 ± 1.10, AB: 2.79 ± 1.09; p=0.652) (Table 1 - see PDF). The overall mean DMFT score was 4.28 ± 2.08. The prevalence of caries experience (DMFT > 0) was 94.8%. The prevalence of high caries experience (DMFT ≥ 5) was 48.5%. Mean DMFT scores differed significantly across blood groups (p=0.002). Participants with blood group O had the highest mean DMFT score (4.82 ± 2.15), followed by B (4.01 ± 2.02), A (3.95 ± 1.98) and AB (3.88 ± 1.85). Post-hoc analysis revealed that the mean DMFT score in group O was significantly higher than in groups A (p=0.004), B (p=0.012) and AB (p=0.008). The prevalence of high caries experience (DMFT ≥ 5) was also significantly higher in blood group O (60.0%) compared to A (41.3%), B (43.3%) and AB (39.6%) (p<0.001) (Table 2 - see PDF). The overall prevalence of gingivitis (GI > 0) was 76.2%. The prevalence of moderate/severe gingivitis (GI ≥ 1.1) was 52.0%. The overall mean GI score was 1.28 ± 0.56. Mean GI scores differed significantly across blood groups (p<0.001). Participants with blood group O had the highest mean GI score (1.42 ± 0.58), followed by B (1.22 ± 0.54), A (1.18 ± 0.51) and AB (1.15 ± 0.49). Post-hoc analysis showed that the mean GI score in group O was significantly higher than in groups A (p<0.001), B (p=0.002) and AB (p<0.001). The prevalence of gingivitis (GI > 0) was significantly higher in blood group O (82.6%) compared to A (71.3%), B (74.0%) and AB (68.8%) (p=0.008). Similarly, the prevalence of moderate/severe gingivitis (GI ≥ 1.1) was highest in group O (63.0%) compared to A (45.3%), B (50.0%) and AB (43.8%) (p<0.001) (Table 2 - see PDF). After adjusting for age, gender, OHI-S score, sugar consumption frequency and tobacco use, blood group O remained a significant independent predictor for both high caries experience (DMFT ≥ 5) and moderate/severe gingivitis (GI ≥ 1.1) (Table 3 - see PDF). Individuals with blood group O had 2.15 times higher odds of having high caries experience (OR=2.15, 95% CI: 1.48-3.12, p<0.001) and 1.94 times higher odds of having moderate/severe gingivitis (OR=1.94, 95% CI: 1.32-2.85, p=0.001) compared to individuals with non-O blood groups (A, B, AB combined). Poor oral hygiene (high OHI-S score) and frequent sugar consumption were also significant independent predictors for both outcomes in the regression models.

## Discussion:

This cross-sectional study investigated the association between ABO blood groups and dental caries and gingivitis among a sample of Indian adults. The key findings revealed a significant and independent association between blood group O and both increased caries experience and higher prevalence and severity of gingivitis, even after adjusting for important confounders like oral hygiene, diet and tobacco use. The observed higher mean DMFT score and prevalence of high caries experience (DMFT ≥ 5) in blood group O individuals align with several previous studies conducted in different populations. A study in Turkey by al-Banyan *et al.* [15] reported significantly higher DMFT scores in blood group O individuals compared to other groups. Similarly, a study among Saudi adults [26] found blood group O associated with a higher risk of dental caries. Conversely, some studies, including one from India by Diaz-Nicolas *et al.* [22], found no significant association between ABO blood groups and caries experience. The reasons for these discrepancies could relate to differences in sample characteristics, age groups, diagnostic criteria, dietary patterns, fluoride exposure and the complex interplay of genetic and environmental factors. The current study's robust methodology, including standardized indices, calibration of examiners and adjustment for confounders, strengthens the validity of the observed association in the Indian context. The biological plausibility for an association between blood group O and caries susceptibility may involve several mechanisms. Salivary composition, particularly the expression of blood group antigens in glycoproteins, can influence the oral microbiome. Individuals with blood group O, especially non-secretors (though not assessed here), may exhibit differences in salivary agglutinins or mucins that could potentially alter bacterial adhesion to tooth surfaces or modulate the host immune response to cariogenic bacteria like Streptococcus mutans [11, 13]. Furthermore, blood group antigens are expressed on various epithelial cells, including those in the oral mucosa, potentially influencing local inflammatory responses and tissue integrity [10]. The finding that blood group O participants in this study reported significantly higher sugar consumption, while adjusted for in the regression, might also suggest a potential behavioral link or interaction warranting further investigation. The significantly higher mean GI score and prevalence of both gingivitis and moderate/severe gingivitis in blood group O individuals a notable finding. This association persisted after adjusting for oral hygiene status (OHI-S), indicating that the increased gingival inflammation in group O was not merely a consequence of poorer plaque control. This result corroborates findings from studies in Iran [17] and Italy [18], which reported worse periodontal health (including gingivitis) in blood group O individuals. However, other studies, such as one conducted in a Brazilian population [20], found no such association. The inconsistency again highlights the potential influence of population-specific genetic and environmental factors.

The link between blood group O and gingivitis could be mediated through inflammatory pathways. Blood group O individuals have been shown to have lower levels of von Willebrand factor (vWF) and factor VIII, which are involved in hemostasis and inflammation [26]. While primarily studied in cardiovascular contexts, altered levels of these factors might theoretically influence vascular permeability and inflammatory cell recruitment in the gingival tissues. Additionally, ABO blood group antigens can modulate the activity of circulating inflammatory cytokines like interleukin-6 (IL-6) and tumor necrosis factor-alpha (TNF-α) [27]. An enhanced or dysregulated inflammatory response to the bacterial biofilm in blood group O individuals could lead to more pronounced gingival inflammation, even at similar levels of plaque accumulation. The observation that tobacco use, a known risk factor for periodontal disease, was also a significant predictor in the gingivitis model, but blood group O remained an independent factor, underscores the potential additive or synergistic effects of genetic and environmental risk factors.

## Conclusion:

A significant association between ABO blood group O and an increased risk of both dental caries and gingivitis among adults in the Indian population is shown. Blood group O individuals exhibited significantly higher mean DMFT scores, a greater prevalence of high caries experience, higher mean Gingival Index scores and a greater prevalence and severity of gingivitis compared to individuals with blood groups A, B and AB. Thus, we show statistically significant even after adjusting for age, gender, oral hygiene status, sugar consumption and tobacco use, suggesting that ABO blood group status is an independent non-modifiable risk factor for these common oral diseases in this population.
